# Observations Respecting an Extraordinary Disease in the Eye of the Horse

**Published:** 1820-08

**Authors:** Henry Atkinson, A. Copland Hutchison

**Affiliations:** Surgeon, at Mangalore, East Indies


FOR THE LONDON MEDICAL AND PHYSICAL JOURNAL.

*Observations respecting an extraordinary Disease in the Eye of the Horse.* [Extract of a Letter from HENRY ATKINSON, Esq. Surgeon, at Mangalore, East Indies, to A. COPLAND HUTCHISON, Esq. &c.]

WE have here a most extraordinary disease among horses, which, I believe, is quite unknown in Europe, either in that animal or in any other: it is a small worm, about one inch in length and the thickness of common silk thread, extremely lively, floating about in the aqueous humour of the eye. After a short time, the humour becomes turbid, milky, opaque; the cornea ulcerates, the humours are evacuated, and the eye is lost. This disease is extremely common; its cause unknown. I should likewise add, that, soon after the eye-ball gives way, the horse gets what we here call weak in the loins; that is, labours under a slight paralysis of the hind-legs, not so much as to prevent locomotion, but sufficient to make it always uneasy, and often dangerous, to ride the horse.

I kept a worm of this sort, which I had taken from the eye of a living horse a few days after the disease appeared: the eye was saved; not even a speck left on the cornea, and the sight perfect. This I did by merely puncturing with the point of a scalpel the transparent cornea at its inferior edge, and about two lines from the sclerotica. I have since performed the operation on several horses with uniform success.

This is a curious disease, and, though in the horse, not divested of great interest; for though found, I believe, only in an inferior animal, still that animal is subject to the same laws of animal life as man; and it may not be impossible that, if men were exposed to the same causes, the like results might follow. Is it impossible that, in the severe purulent ophthalmia of Egypt and of India, that this worm may not exist, and be its immediate cause? In the horse it is sometimes with difficulty discovered, even when looked for: in man, where it is not looked for, it may, from this and other causes, have escaped notice. This I do not give as an opinion, for I do not believe it to be so; but merely as a curious speculative question.

*Remarks on the above Observations.* By A. C. HUTCHISON, Esq.

THE disease described by Mr. Atkinson I have seen noticed, within these two years, in some one of the periodical Journals; but Mr. A.'s account is much more interesting, because in it we have the method of cure detailed. The suggestion he has thrown out, as to the probable cause of Egyptian ophthalmia in man, is very ingenious, and highly deserving of notice; and must lead, I think, to the most careful dissection of the eye after the destruction of sight by that disease, when opportunities offer.

One can hardly account for the partial paralysis of the hind legs of the horse, as a consequence of this disease, unless it be the irritation produced by the worm in its various movements, &c. within the eye, and the irritation thence communicated to the brain by the optic and other nerves serving that organ.

I have written to Mr. Atkinson, to send me home some of the worms he has noticed, that their species may be examined into; when I shall not fail to communicate to the profession any farther particulars that I may be furnished with, or that may be interesting to science.

57, *Spring Garden; July* 1820.

